# Activities, Participation and Quality of Life Concepts in Children and Adolescents with Celiac Disease: A Scoping Review

**DOI:** 10.3390/nu9090929

**Published:** 2017-08-24

**Authors:** Sonya Meyer, Sara Rosenblum

**Affiliations:** The Laboratory of Complex Human Activity and Participation, Department of Occupational Therapy, Faculty of Social Welfare & Health Sciences, University of Haifa, Haifa 3498838, Israel; rosens@research.haifa.ac.il

**Keywords:** Celiac disease, gluten-free diet, ICF-CY, food-related activities, quality of life

## Abstract

Celiac disease (CD) is a food-related chronic condition and adherence to a strict gluten-free diet is the only available treatment. Adherence to the restrictive diet is challenging among children, especially adolescents. The aim was to describe existing knowledge about food-related activities, participation, and quality of life in daily life among children and adolescents with CD and to illuminate gaps in knowledge. The scoping review methodology was applied and literature searches were conducted in electronic databases. Twenty-three articles met the inclusion criteria. Food-related activities were identified, classified, and coded under the International Classification of Functioning, Disability, and Health adapted for children and youth (ICF-CY) concepts of activities and participation. A wide variety of study populations, objectives, methods, and tools involving 55 different food-related activities were found. Incorporation of the ICF-CY concepts and quality of life captures new insights into everyday challenges. Reviewing the CD literature using this different lens reveals areas yet to receive sufficient attention. Further research can deepen the understanding of daily functioning of children with CD and the underlying skills required to participate in daily food-related activities while adhering to the diet. This can lead to the development of standardized disease-specific assessment tools and suitable intervention programs.

## 1. Introduction

Celiac disease (CD) is a genetic food-related chronic condition that is precipitated by exposure to gluten [1]. Its prevalence varies from 0.3% to 1.3% of the population and its recognition as a public health problem is increasing [2]. A lifelong adherence to a strict gluten-free diet (GFD) remains the only known effective treatment [3].

Adherence to the restrictive GFD is vital for health outcomes, however, it entails complete avoidance of gluten-containing foods and is a challenge among children, especially adolescents with CD [4,5]. As children with CD grow, they face new challenges, such as social pressure, increasing independence from their parents, and new responsibilities for various food-related activities. These challenges can lead to newfound difficulties in adhering to the diet, especially in social settings [6,7]. 

**Figure 1:**
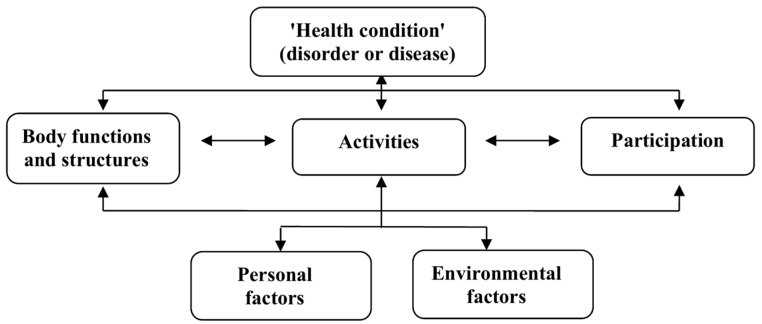
The International Classification of Functioning, Disability and Health: Children and Youth Version [8]. Reproduced with permission of the World Health Organization.

This review will describe existing CD literature focused on the concepts of activities and participation that appear in the International Classification of Functioning, Disability, and Health (ICF) [8] and the version for children and youth (ICF-CY) [9] frameworks. The ICF describes interactions between activities and participation, body functions, and structures while considering both environmental and personal factors [8] (Figure 1).

With the introduction of these concepts, the concept of health has broadened to a multidimensional construct that encompasses life′s psychological and social dimensions, as well as its biomedical constituents [10]. The ICF serves as a common language among health professions. Applying the ICF concepts in the clinical evaluation and intervention process has the potential to promote the participation, inclusion experience, and well-being of people with chronic conditions [11,12]. 

The ICF-CY defines activity as the execution of tasks and participation is the involvement in daily life situations [9]. Activity limitations are difficulties an individual with a specific health condition may have when executing and experiencing activities in comparison to those without the condition [9]. Participation restrictions are problems an individual may experience in involvement in life situations [9]. Focusing on activities and participation concepts may allow better understanding of the everyday challenges of children and adolescents with CD [13]. 

Participation among children is often discussed in the literature together with quality of life (QOL) and its component, health-related quality of life (HRQOL), which focuses specifically on the QOL influenced by a health condition [14,15]. HRQOL represents a multidimensional construct which evaluates physical, psychological, social, and cognitive components [14]. Research and exploration of QOL and HRQOL of children coping with CD have received increased attention over the last decade, increasing in recent years (e.g., [16,17,18,19]). The ICF includes extensive aspects of functioning disability and health and contains items included that are in QOL and HRQOL measures. Thus, it can elucidate various health related concepts, including QOL and HRQOL [20]. Therefore, QOL and HRQOL concepts will be incorporated in the current review and referred to, inclusively, as QOL [14].

Activity limitations for children and adolescents with CD result from the need to avoid gluten-containing foods while participating in life situations. For some the GFD is simple yet, for others, maintaining the diet in daily life can be challenging and may present participation limitations for them and their families [21,22]. White and colleagues [21] reviewed the factors associated with adherence and HRQOL among adolescents with CD. Various burdens were highlighted, including challenges when eating out, travelling, and socializing with friends, which were associated with poor adherence to the GFD and HRQOL. Emotional support and stronger organization skills were associated with better adherence. Although vital aspects were discussed, the focus was not specifically on activities and participation. 

In light of the ICF-CY [9], the purpose of this scoping review is to describe existing knowledge concerning food-related activities and participation and QOL in daily life among children and adolescents with CD and to illuminate gaps in knowledge that may lead to future research.

## 2. Materials and Methods

The scoping review method was chosen for this article. This methodology addresses an exploratory research question aimed at mapping key concepts and gaps in evidence related to a defined field by systematically searching, selecting, and synthesizing knowledge [23,24].

### 2.1. Identifying the Research Question

The question that guided this scoping review was, what is known about the ICF activities and participation concepts and QOL in the specific context of food-related activities among children and adolescents with CD concerning daily food-related activities?

### 2.2. Identifying Relevant Studies and Study Selection

**Figure 2:**
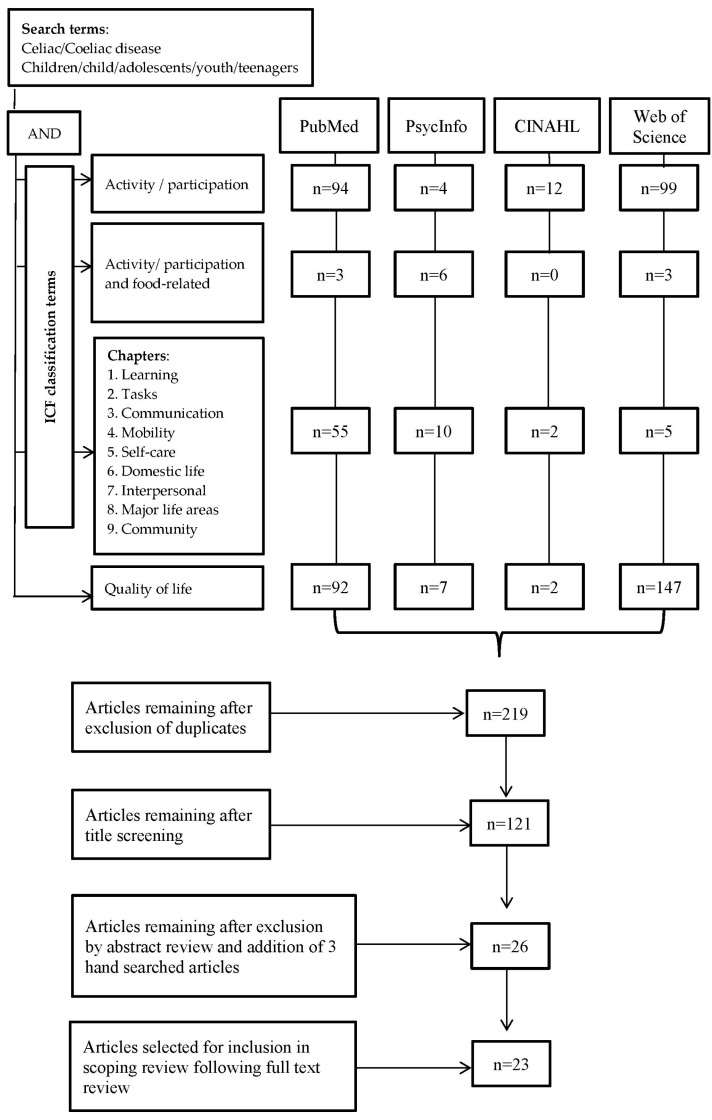
Flow diagram describing the search and inclusion process.

A thorough search of the literature was conducted using the databases PubMed, CINAHL, PsycINFO, and Web of Science. Figure 2 outlines the data gathering and elimination procedures and the study selection process.

Initially, the keywords “Celiac or Coeliac disease” AND “children OR adolescents OR child OR youth OR teenagers” were entered in each data base and then “activity” “participation” using the “AND/OR” Boolean connectors. Thereafter, keywords representing each of the nine activities and participation ICF-CY chapters [9] and “quality of life” were added one at a time using the Boolean connector “AND” (Figure 2).

### 2.3. Study Selection

The eligible inclusion criteria were English articles published in peer-reviewed journals, between January 2006 and June 2016. Publications were included if they focused on children and adolescents with CD and referred to any aspect of food-related activities and participation in daily life with CD. Publications that concentrated only on adherence to the GFD were excluded, as this is not the focus of the review. Three articles that did not appear in the described search process were added. Two articles were found relevant due to extensive previous literature exploration and one was an article written by the authors of this review but yet to be indexed at the time of the search process. These articles are marked with an asterisk in Table 1. The first author screened the titles, abstracts, and full texts of articles corresponding with inclusion criteria while consulting with the second author throughout the entire process. The authors discussed and deliberated the selection of the papers by mutual agreement. After final selection, the full articles were independently read by a research assistant, knowledgeable about the ICF-CY framework. Expert agreement was obtained regarding the relevance of the selected articles to this scoping review and regarding the inclusion of articles.

### 2.4. Charting the Data

Information from each article was independently recorded by the first author and the research assistant. Records included the authors, publication year, diagnosis, age of subjects, participants, study objectives, methods, tools, and ICF-CY classification of the identified food-related activities. There was agreement between the authors concerning most of the charted data. The few disagreements were discussed between the authors and the research assistant and consensus was obtained. The activities and participation ICF-CY classification is created by linking information to an alphanumeric coding system, thus organizing the health information in a classification comprised of up to four levels, where each level is more detailed and specific than its previous level [9,12]. The activities and participation component is coded with the letter d and its first-level classification includes nine chapters that are coded from d1 to d9; d1, learning and applying knowledge; d2, general tasks and demands; d3, communication; d4, mobility; d5, self-care; d6, domestic life; d7, interpersonal interactions and relationships; d8, major life areas; and d9, community, social, and civic life. The second-level consists of more detailed classifications within each of the nine chapters, defined as categories that are coded from d110 to d999. Later, food-related activities identified in the reviewed articles were linked to first and second-level ICF-CY activities and participation codes [9]. According to the ICF, QOL concepts are integrated within the ICF terminology [20]. Therefore, QOL principles derived from the reviewed articles will all be presented by means of the ICF-CY [9] terminology. 

## 3. Results

The search strategy initially yielded a total of 658 articles (Figure 2). After a full text review, four of the articles were excluded, of which three articles included heterogeneous samples and one was not specific to food-related activities. The review process ultimately included 23 articles that met the criteria for the final data extraction and are described in Table 1.

### 3.1. Study Populations

Children and adolescents with CD aged two to 18 years old and/or their parents participated in the studies. In 14 of the 23 articles children and/or adolescents and their parents participated [13,16,18,25,26,27,28,29,30,31,32,33,34], in seven studies children and/or adolescents participated [35,36,37,38,39], and in one article only parents participated [40] (Table 1).

### 3.2. Study Objectives

The 23 articles included five main objectives, as follows: (1) Twelve articles involved assessment of QOL [13,16,19,26,29,31,32,33,35,36,37,42]; (2) Seven articles were associated with adherence to the GFD [28,35,36,39,41]; (3) Six articles were associated with the impact of a GFD on daily life [18,27,33,38,43]; (4) Five of the 23 articles were related to the impact of CD on the family or parents [25,29,38,39,40]; and (5) Five of the articles explored the relationships of QOL and adherence [13,28,35,36,41].

### 3.3. Study Methods and Tools 

A variety of research methods and tools involving the food-related activities were reported in the 23 reviewed articles. One article was a review article [41] and qualitative methods were utilized in eight of the articles including three interview studies [25,32,40], of which three used focus groups [33,38,43], one used open-ended questions [18], and one used recorded conversations [34]. Food-related activities and participation were assessed by various quantitative questionnaires in the remaining 14 reviewed articles. 

A range of disease-specific questionnaires in which the food-related activities identified were developed, implemented or validated. The celiac disease DUX (CDDUX), a disease-specific health-related quality-of-life questionnaire for children with CD, developed in The Netherlands to assess both self-report and parental report of health-related QOL [16], has been translated and is in use in four additional countries [13,26,31,42]. Six of the articles used self-report questionnaires developed specifically for the purpose of the study [28,30,36,37,39]. In two additional articles, CD specific questions were added to generic self-report QOL questionnaires [27,36]. One article used a non-disease-specific QOL questionnaire [29] and one article implemented a participation in leisure activities questionnaire [13]. 

The constructs measured in the different questionnaires also varied. Seven of the articles used frequency ratings of avoidance, difficulty, and negative feelings, such as anger and embarrassment [19,27,28,30,35,37,39]. Five articles, which utilized the same questionnaire, used a scale that rated feelings concerning CD on a scale of a very good to a very bad feeling [13,16,26,31,42]. One article used a non-disease-specific questionnaire that rated QOL on a scale of very unhappy to very happy [29]. One article included the food-related activities in a questionnaire that incorporated a range of CD related questions, each represented by different response formats [36]. Meyer and Rosenblum [13] rated the participation in food-related activities in terms of the frequency of participation, with whom the activity was performed and how much the child liked it.

### 3.4. Activities and Participation in Food-Related Activities

Only one article made reference to the defined ICF-CY activities and participation concepts [13]. Nevertheless, a variety of food-related activities and participation restrictions were identified in all of the 23 articles. These activities were classified according to the ICF-CY first-level activities and participation chapter codes, and then second-level category codes [9] (Table 2). 

#### 3.4.1. Community, Social, and Civic life

A total of 23 different food-related activities identified in 17 articles were classified within the first-level community, social, and civic life chapter (Table 2). The second-level classifications were mostly coded under the recreation and leisure category [9] (Table 2). Participation in ‘social functions/events’, ‘parties/birthday parties’, ‘eating at restaurants’, and ‘eating/dining out with friends’ were the most frequently discussed activities in this category, followed by ‘travelling’, ‘eating at a friend’s house’, and ‘vacations’. The remaining food-related activities in this category were only mentioned once each in three different articles (Table 2). The last two activities in this chapter, ‘summer camp’ and ‘sports camp’, were coded in the community life category and were mentioned in two different articles (Table 2).

#### 3.4.2. Major Life Areas

The following group included 11 different food-related activities that were mentioned in all of the articles and classified in the first-level major life areas chapter within a sub-chapter of education [9] (Table 2). These activities were all coded in the second-level school life and related activities category [9]. The most frequently mentioned food-related situations in school life were defined as ‘food situations/activities/events at school’ which were the topics mentioned in seven of the articles (Table 2). The following were ‘given gluten food at school’ and ‘eating at school cafeteria/canteen’. Four articles mentioned ‘meals or eating at school’ and ‘eating with friends at school’ was mentioned in two of the articles. The remaining food-related activities in this category were mentioned only once each in four different articles (Table 2).

#### 3.4.3. Self-Care

Ten different food-related activities found in 10 of the articles were classified in the first-level self-care chapter (Table 2) and classified mostly in the second-level ‘looking after one’s health’ category. These activities appeared in six articles and included ‘being offered gluten food’, ‘thinking of gluten food’, ‘not being able to eat anything’, ‘following a lifelong diet’, ‘paying attention to what I eat’, ‘having CD’, ‘not eating what others eat’, and ‘following a diet for my CD’. Five of these articles described the development of the CDDUX [16], as well as translations and cultural implementation of the questionnaire [13,26,31,42]. Additional activities coded in the ‘looking after one’s health’ category are detailed in Table 2. 

#### 3.4.4. Communication

Items in the CDDUX communication scale, that encompassed three food-related activities including ‘talking about CD’, ‘explaining about CD’ and ‘talking about CD to friends’, were coded in the first-level communication chapter and second-level category ‘conversation’. These activities appeared in five of the articles that involved the use of this this questionnaire [13,16,26,31,42] (Table 2).

#### 3.4.5. Domestic Life, Interpersonal Interactions, and Relationships and General Tasks and Demands

Two food-related activities concerning shopping for gluten-free food were classified under the domestic life chapter and two other food-related activities describing relationships with teachers were classified in the interpersonal interactions and relationships chapter (Table 2). Finally, two food-related activities occurring in the regular home schedule were classified in the general tasks and demands chapter (Table 2).

## 4. Discussion

In this scoping review, food-related activities and participation of children and adolescents with CD in everyday life were identified within the CD literature via the lens of the ICF-CY concepts [9], as well as their QOL. The review displayed that although the research on children and adolescents with CD is expanding, there are still new avenues to be explored. By focusing specifically on activities and participation, a wide range of food-related activities emerged alongside two significant literature gaps. 

The first, is the wide disparity in the study populations, objectives, methods and tools. The age of the study population in the reviewed articles, ranged from two to 18 years, and each age group has distinct developmental characteristics. Development involves considerable changes from birth to adolescence while achieving increasingly complex skills involving actions and reactions to both their physical and social environment [44]. During this dynamic process, the child transitions from dependency in infancy towards physical, social, and psychological maturity and independence in adolescence [9]. In the context of CD specifically, children and adolescents are faced with new challenges throughout childhood [6,21,45]. Accordingly, the daily needs, coping characteristics and activities in which the children and adolescents participate differ immensely and each age group needs to be addressed according to its developmental stage. 

Disparity is also apparent in the varied study objectives and consequently, in the assortment of study methodologies. Vast differences were revealed in the types of qualitative methods implemented and quantitative questionnaires used. The CDDUX [16] was the most frequently used tool and was implemented in a number of different languages [13,26,31,42]. Other studies added disease-specific questions to existing generic measures or developed new measures [19,27,28,35,37,39], thus stressing the need for standardized tools that capture and reflect the unique characteristics of life with CD. In some studies children and adolescents were either interviewed or completed self-report questionnaires while, in others, parents were the ones providing the responses, and, thus, they reflected different perspectives. When measuring children’s participation both the child’s and parents’ perspectives are of relevance [46]. Parents of children with CD tend to rate their children′s HRQOL lower than the children’s own ratings [47]. Nevertheless, the contribution of children’s and adolescents’ self-reports as the most valid approach to learning about their HRQOL has been established and the importance of hearing children′s self-perception is underlined [16,48]. Children′s perceptions of how healthcare impacts their QOL have a direct effect on the ability to improve their QOL [49]. Therefore, the incorporation of self-reports into research of children and adolescents with CD is essential to understanding their daily life. Due to all the above-mentioned aspects of diversity, the comparison and generalization of results found in this review are limited.

Given the importance and the significant presence of food-related issues during the daily life with CD, a second gap was identified. The interpretation given to the food-related activities found in the literature and their unique meaning in the daily life to those with CD is lacking. The literature review exposed a total of 55 food-related activities that spread across seven of the nine one-level ICF-CY activities and participation chapters. Thus, the extent of their presence is clearly reflected in both daily functioning and in experiences of involvement in daily life situations of children and adolescents with CD. However, the meaning in most of the reviewed articles was not specifically on activities and participation limitation analysis and did not reflect the full scope implications of food-related daily activities. Overall, the information obtained from these tools concentrated mainly on the negative emotions experienced and the frequency of problems experienced. The utter nature of the participation component is the inclusion of both attendance and involvement [50]. Therefore, in order to fully understand the meaning of participation in these and other food-related activities to the children and adolescents themselves, in the context of CD, there is need to consider additional aspects that extend beyond going to the activity and the emotions that arise. The ICF-CY constructs and components can assist in directing health professionals to ask the questions required to obtain a comprehensive understanding of what is important to the child’s or adolescent’s daily functioning. 

The majority of food-related activities in the review were categorized under two chapters. The first chapter is community, social, and civic life, and especially recreation and leisure [9]. Among all children participating in activities that are performed with others, having fun, and experiencing a sense of control promotes their sense of confidence and feeling of well-being [51]. Furthermore, participation plays a key role in children’s development, particularly in leisure activities outside of school, and provides the context within which children and young people develop and prepare for life transitions [11]. The second chapter is major life areas and particularly school life [9]. School years, including both academic and nonacademic education, are a significant part of childhood that lasts for over a decade [52,53]. Hence, information about the activities and participation supports and limitations among this population is imperative to fully understand their daily confrontations in community, social, and civic life, and in major life areas, such as school. Indeed, previous primary analysis of the relationship between these factors revealed that the better children with CD feel with their health condition, so they participate in food-related activities with a higher social level, meaning with a greater number of friends [13]. Given such insights, there is a need to deepen the understanding of the various aspects of daily life beyond rating the level of adherence and observe research outcomes in-depth and beyond mean scores [54]. Paucity was found is reference to the different practical activity demands required for participation in specific food-related activities. For example, what kind of preparation is required before participation in the food-related activities, who is responsible for the preparation, and how involved the child or adolescent is in the preparation process. 

This scoping review has several limitations. First, there is always the possibility that literature may have been missed despite the meticulous search strategy and process. Second, this study was limited to four databases. Searching additional databases might have resulted in more articles to review, yielding additional food-related activities. Third, a certain level of subjectivity is unavoidable in the decision-making process when considering inclusion and exclusion of studies and in conducting the ICF-CY analysis. To address this limitation, decisions were made by the authors and a research assistant. Finally, differences in the clinical symptomology, which would be defined by the ICF as body functions, were evident in reviewed articles. Additionally, demographic variables, defined by the ICF as personal and environmental factors, were diverse. This review did not focus on these ICF components. However, as the ICF describes multidirectional interactions between its components [8] (Figure 1), further exploration of these variables could be beneficial in providing information that can impact activities and participation and QOL. 

## 5. Conclusions

This scoping review mapped out the literature on food-related activities and participation of children and adolescents with CD. A wide range of food-related activities occurring in the daily lives of this population were identified within the literature. However, these food-related activities are not presented in the literature in the context of the ICF-CY, nor do they receive the in-depth analysis as provided by this classification. The complexity of understanding of each activity, its demands, limitations, challenges, and its importance to each child and adolescent with CD is limited. Consequently, important information concerning the actual daily life of this population can be overlooked. This review demonstrated that incorporation of the ICF-CY [9] concepts of activities and participation and QOL can capture everyday challenges through a different lens than they were originally seen through. This different view can enable identification and organization of information and issues that are important to children and adolescents with CD and, thus, identify areas yet to receive sufficient attention. Future research, linked to the ICF-CY constructs [9] can enhance comprehension of how daily life activity limitations impact participation. Further ICF-based knowledge on the subject may lead to the development of disease specific standardized assessment tools and intervention programs that consider the daily functioning of children with CD.

## Figures and Tables

**Table 1:** Articles included in the scoping review.

Author/s	Key Words	Participants	Ages in Years	Type of Study	Constructs Measured
Altobelli et al., 2013 [35]	N/A	*n* = 140	10–18	Quantitative	CD specific questions-frequency of negative feelings (e.g., feel angry, bad, embarrassed),
Arnone and Fitzsimons, 2012 [41]	N/A	Adolescence		Review	Psychological aspects of CD
Bacigalupe and Plocha, 2015 [25]	adherence; CD; family rituals; GFD; social support	*n* = 10 (children and parents)	6–12	Qualitative interviews	Family challenges and strategies
Barrio Torres et al., 2016 [26]	disease-specific questionnaire; outcome health; transcultural adaptation	*n* = 214 (parents only), *n* = 214 (parents and children), *n* = 52 (children only)	10–18	Quantitative	HRQOL
Bellini et al., 2011 [36]	N/A	*n* = 156 (CD), *n* = 353 (healthy controls)	6–16	Quantitative	QOL scale (e.g., feeling embarrassed; feeling unhappy giving up some group activities due to temptation not to follow the GFD)
Biagetti et al., 2013 [18]	QOL; CD; GFD; children; lived experiences; psycho-social aspects	*N* = 76 >8 years (children) <8 years (parents)	2–18	Qualitative open ended questions	Emotional impact of GFD on everyday life
Biagetti et al., 2015 [27]	Child; GFD; HRQOL	*n* = 76 (CD); *n* = 143 (non-CD)	2–18	Quantitative	QOL; impact of GFD on social life
Bongiovanni et al., 2010 [37]	Pediatric; sprue; QOL	*n* = 77	7–17	Quantitative	General well-being; emotional outlook; self-perception (e.g., difficulty doing)
Cederborg et al., 2011 [40]	adaption process; children; coeliac disease; parental perspective	*n* = 20 (children and parents)	3–5; 16–17	Qualitative interviews	Parental perspective of living with CD
Chauhan et al., 2010 [28]	CD; GFD; compliance; QOL	*n* = 70	2–17	Interview consisted of a self-administered questionnaire	Effect of CD on child′s feeling
de Lorenzo et al., 2012 [29]	CD; QOL; child; parents	*n* = 33 (CD), *n* = 63 (non-CD), *n* = 96 (parents)	5–12	Quantitative	Leisure
Jordan et al., 2013 [19]	CD; children; focus groups, GFD; HRQOL	*N* = 181	8–18	Quantitative	HRQOL
Lins et al., 2015 [42]	QoL; Cross-cultural adaptation; CD	*n* = 33 (children and parents)	8–18	Quantitative	HRQOL
MacCulloch and Rashid, 2014 [30] *	Adherence; CD; compliance; GFD	*n* = 126	2–18	Quantitative	HRQOL
Meyer and Rosenblum, 2016 [13] *	CD; child; leisure activities; parents; QOL; self-report	*n* = 34 (CD), *n* = 34 (healthy controls)	8–15	Quantitative	HRQOL, Leisure
Olsson et al., 2008 [43]	Adolescent; CD; focus groups; GFD; patient compliance	*n* = 57	15–18	Qualitative focus groups	Views of everyday life with coeliac disease and a prescription of a GFD
Olsson et al., 2009 [38]	Adolescent; chronic illness; focus groups; lived experiences; social constructionism; stigma	*n* = 57	15–18	Qualitative focus groups	The GFD can produce stigma experiences in adolescence
Pico and Spirito, 2014 [31]	QOL, children/adolescents, CD, CDDUX, sensitivity to change	*n* = 118 (children and parents)	8–18	Quantitative	HRQOL
Roma et al., 2010 [39]	Children; CD; compliance; GFD; life style	*n* = 73 (self-report and parents)	5–14.5	Quantitative	HRQOL
Rosén et al., 2011 [32]	N/A	*n* = 101 (adolescents) *n* = 125 (parents)	13.9–15.4	Qualitative interviews	
Skjerning et al., 2014 [33]	CD; HRQOL; Children; Adolescents/youth; Coping; focus groups; illness and disease; chronic	*n* = 23 (children/adolescents) *n* = 3 (parents)	8–18	Qualitative focus group interviews	HRQOL
van Doorn et al., 2008 [16]	CD; QOL; disease specific questionnaire; proxy	*n* = 530	8–18	Quantitative	HRQOL
Veen et al., 2012 [34] *	Discursive psychology, coeliac disease, family mealtime, discourse analysis, experience of illness	*n* = 7	2–20	Qualitative	Conversation about the food during meals

* = hand searched articles; N/A = not available; CD = celiac disease; CDDUX = celiac disease DUX; GFD = gluten-free diet; QoL = quality of life; HRQoL = health related quality of life.

**Table 2:** Activities and participation first- and second-level ICF classification.

ICF Classification	Food-Related Activities	*n*
d9 Community, social, and civic life		
d920 Recreation and leisure	Eating at restaurants	8 [13,19,30,33,35,36,38,40]
	Parties/birthday parties	7 [25,27,28,29,32,38,43]
	Social functions/events	7 [19,25,28,33,37,38,40]
	Eating/dining out with friends	6 [28,32,33,38,41,43]
	Travelling	4 [28,30,35,43]
	Eating at a friend’s house	3 [19,36,38]
	Vacations	2 [29,33]
	Once each: Picnics; visiting people/family [13]; activities at friends; bringing GF food when traveling [35]; group activities [37]; meals outside the home with peers; social engagement outside the home; communal meals with peers [41]; sleep overs [25]; eating meals outside home [18]; eating GF food in public [38]; travelling abroad; bringing GF food to a meal; spontaneous social life with friends [40]	1
d910 Community life	Summer camp	2 [25,43]
	Sports camp	1 [43]
d8 Major life areas-education		
d835 School life and related activities	Food situations/activities/events at school	7 [18,25,29,35,38,39,41]
	Given gluten foods at school	5 [13,16,26,31,42]
	Eating at school cafeteria/canteen	4 [27,32,38,39]
	Meals/eating at school/daycare	4 [30,40,43]
	Eating with friends at school	2 [19,29]
	Once each: school parties; after-school activities [19]; unexpected snack in class; play games with food in class [33]; eating pastries in class [43]; home economic classes [38]	1
d5 Self-care		
d570 Looking after one’s health	Not being able to eat anything/ Paying attention to what I eat/ not eating what others eat/ frustrated about not eating something you want	6 [13,16,26,31,37,42]
	Following a lifelong diet/ following a diet for my CD/angry about having to follow a special diet	6 [13,16,26,31,37,42]
	thinking of gluten food	5 [13,16,26,31,42]
	Offered gluten food	5 [13,16,26,31,42]
	Having CD	5 [13,16,26,31,42]
	Once each: meals at home [18]; knowing what to eat; making sure there is GF food before visiting friends [40]	1
d550 Eating	Eating with the family	2 [19,29]
	Having meals	1 [29]
d3 Communication		
d350 Conversation	Talking about CD	5 [13,16,26,31,42]
	Explaining about CD	5 [13,16,26,31,42]
	Talking about CD to friends	5 [13,16,26,31,42]
d6 Domestic life		
d620 Acquisition of goods and services	Determining if food is GF or not from the food label	2 [19,35]
	Finding GF food in stores	1 [35]
d7 Interpersonal interactions and relationships		
d740 Formal relationships	Feel teachers do not understand	2 [28,35]
	Talking to school staff	1 [40]
d2 General tasks and demands		
d230 Carrying out daily routine	Once each: eating at home [40]; cooking at home [43]	1

*n* = incidence in which the food-related activities are mentioned in the reviewed articles; CD = celiac disease; GF = gluten free; ICF = International classification of functioning, disability, and health.
